# Cerebral Protection Strategies in Aortic Arch Surgery—Past Developments, Current Evidence, and Future Innovation

**DOI:** 10.3390/bioengineering11080775

**Published:** 2024-07-31

**Authors:** Paul Werner, Martin Winter, Stephané Mahr, Marie-Elisabeth Stelzmueller, Daniel Zimpfer, Marek Ehrlich

**Affiliations:** Department of Cardiac Surgery, Medical University of Vienna, Waehringer Guertel 18-20, 1090 Vienna, Austria; stephane.mahr@meduniwien.ac.at (S.M.); marie-elisabeth.stelzmueller@meduniwien.ac.at (M.-E.S.); daniel.zimpfer@meduniwien.ac.at (D.Z.); marek.ehrlich@meduniwien.ac.at (M.E.)

**Keywords:** aortic arch surgery, cerebral protection, cerebral perfusion, hypothermic circulatory arrest

## Abstract

Surgery of the aortic arch remains a complex procedure, with neurological events such as stroke remaining its most dreaded complications. Changes in surgical technique and the continuous innovation in neuroprotective strategies have led to a significant decrease in cerebral and spinal events. Different modes of cerebral perfusion, varying grades of hypothermia, and a number of pharmacological strategies all aim to reduce hypoxic and ischemic cerebral injury, yet there is no evidence indicating the clear superiority of one method over another. While surgical results continue to improve, novel hybrid and interventional techniques are just entering the stage and the question of optimal neuroprotection remains up to date. Within this perspective statement, we want to shed light on the current evidence and controversies of cerebral protection in aortic arch surgery, as well as what is on the horizon in this fast-evolving field. We further present our institutional approach as a large tertiary aortic reference center.

## 1. Introduction

Aortic arch surgery is considered a demanding procedure for the patient and potentially also for the surgeon. Continuous efforts to achieve better outcomes have advanced the field of arch operations within the last decades. Nevertheless, it remains a high-risk surgical procedure with relevant perioperative mortality and morbidity [1]. Cerebral injury is one of the major contributors to operative morbidity, with rates of permanent neurological deficit ranging from 3 to 8% in elective cases, and up to 18% in emergent (Type A Dissection) surgery [2,3].

## 2. Cerebral Perfusion Modes

In 1975, the pioneer Randall Griepp was the first one to cool a patient to 18 °C and establish deep hypothermic circulatory arrest (HCA) to replace an aneurysmatic aortic arch, offsetting a long trajectory of improvements in order to keep the brain protected while operating on the arch [4]. Deep hypothermic circulatory arrest (DHCA) was limited to relatively short time intervals; therefore, additional measures to increase circulatory arrest times were required. Retrograde cerebral perfusion (RCP), primarily developed to treat massive cerebral air embolism in 1980, was introduced in arch surgery to establish blood flow to the brain via the superior vena cava by Ueda and colleagues in 1990 [5,6]. Retrograde cerebral perfusion has been proven to lower operative mortality and decrease permanent neurologic complications during longer HCA times [7,8]. However, a definite mechanism of cerebral protection during RCP other than continuous and homogenous cooling are still a matter of discussion [9]. Antegrade cerebral perfusion (ACP) was introduced to arch surgery around the same period as RCP in parallel by two groups from Japan and France [10,11]. In comparison to RCP, ACP mimics more of the physiologic conditions of cerebral blood flow. Deep hypothermic cardiac arrest with antegrade cerebral perfusion significantly reduced operative mortality and allowed longer circulatory arrest (CA) times, giving rise to more complex procedures on the aortic arch [12]. Initially, RCP and ACP were still used in combination with deep hypothermia and, thus, were associated with coagulopathy, long rewarming, and organ dysfunction. Therefore, moderate (20.1–28 °C) to mild- (28.1–34 °C) hypothermia combined with antegrade cerebral perfusion was introduced and proven to be a safe method to protect both the brain and visceral organs during aortic arch surgery [13,14]. A currently discussed topic is whether ACP should be established via a uni- or bilateral approach. In bilateral ACP, blood flow to the brain is ultimately provided via both carotid arteries, leading to the perfusion of both hemispheres, which resembles the physiologic state. However, the fact that two cannulas must be introduced to achieve bilateral perfusion also causes the further manipulation of aortic arch vessels, which leads to concerns regarding higher risks of embolism during bi-ACP [15,16]. Numerous cannulation strategies for uni- and bilateral ACP (right axillary artery, common carotid arteries, innominate artery) have been implemented but there has been no clear consensus favoring one access route over the others [17]. Several single- and multi-center studies investigating cerebral perfusion strategies reported contradicting results. In a propensity-matched retrospective study by Piperata et al. with patients with acute type A aortic dissection (ATAAD), a significantly lower incidence of neurologic complications was observed with unilateral ACP versus bilateral ACP [18]. In contrast, a retrospective study investigating ACP in ATAAD from 2020 concluded that bilateral ACP increases overall survival when ACP exceeds the time limit of 50 min [3]. Due to a lack of prospective randomized trials, the question of superiority of one strategy remains open, with the best evidence coming from large registries. An analysis from the German Registry for Acute Aortic Dissection Type A (GERAADA) reported similar outcomes for HCA and HCA with ACP for circulatory arrest times under 30 min, but observed a significantly increased mortality when HCA exceeded times of 30 min; therefore, they recommended the use of ACP in these circumstances [19]. No differences in neurological deficits and mortality were found between uni- and bilateral ACP. Similar results were found in an analysis form the STS database, confirming HCA as a safe option when circulatory arrest times remain short [20]. Nevertheless, it remains essential to anticipate which patients will require longer CA times and more complex aortic repairs because these patients benefit from adjunctive cerebral perfusion such as RCP and ACP. In a nationwide study from Japan that included 16,218 who underwent total arch replacement between 2009 and 2012, HCA + RCP compared to ACP had similar mortality and neurologic outcomes, although the incidence of postoperative ICU stays was significantly higher in the RCP group [21]. The most recent results from the UK National Adult Cardiac Surgical Audit showed a lower risk of death for uni- and bilateral ACP compared to DHCA. The authors concluded that unilateral ACP might be considered for short CA times, while patients with longer CA times might benefit from the use of bilateral ACP [22]. Table 1 summarizes relevant studies investigating the effects of different perfusion techniques and temperatures on both temporary and permanent neurological deficits after aortic arch repair.

## 3. Temperature Management

The main rationale for introducing DHCA in arch surgery was to decrease the metabolic rate of the brain and other organs and, therefore, to increase ischemic tolerance while operating on the aortic arch and maintaining a bloodless operative field. The use of ACP created the question of whether extended hypothermia is still necessary for cerebral protection. Subsequent studies evaluating this question revealed that the use of ACP with mild to moderate hypothermia is a safe option that provides good organ protection during circulatory arrest [14,24]. In a series of 544 patients undergoing aortic arch surgery, Preventza et al. assessed the outcomes of patients with different degrees of temperatures during ACP. No statistically significant difference regarding short-term mortality and major morbidity was found between the deep, low-moderate, and high-moderate hypothermia groups, although they observed a trend towards a higher incidence of adverse events in the deep hypothermia group [25]. When analyzing the actual temperatures continuously, a statistically significant reduction in stroke rates, as well as lower rates of reoperations and bleeding, was observed with higher temperatures.

This issue was also examined by Tian et al. in a large meta-analysis of 18 studies and 2632 patients undergoing aortic arch surgery using antegrade cerebral perfusion. They divided patients into two groups: the mean temperature was 20.3 °C in the “cold” group and 26.5 °C in the “warm” group. Briefly, they concluded that higher temperatures during circulatory arrest may reduce permanent neurologic deficits, and they observed higher mortality in the lower temperature group. When comparing the different degrees of temperatures separately, they found a significantly higher mortality in the deep-hypothermia group when compared to the moderate-hypothermia group. However, the comparison of low-moderate and high-moderate hypothermia as well as moderate hypothermia and mild hypothermia did not reach statistical significance. They did observe a trend of lower mortality with higher temperatures [26]. A recent meta-analysis, evaluating 34 studies and 12,370 patients undergoing aortic arch surgery, observed an increased risk of neurological complications with DHCA as well as higher operative mortality. This study did not exclusively investigate temperatures during ACP, i.e., it included patients receiving RCP in the setting of DHCA, and ACP was used mainly in patients with mild HCA. However, these trends of higher temperatures being associated with fewer complications in such a large series of patients is a relevant finding [27]. In summary these observations of better outcomes with higher temperatures using ACP reveal a certain trend.

In contrast to those findings, a recent randomized single blind trial (GOT ICE) evaluated the effects of different levels of hypothermia on cognitive function 4 weeks after aortic arch surgery. There was no difference between the groups except that verbal memory was significantly better in the deep hypothermia group compared to moderate [28]. Ultimately, large randomized prospective trials are required to establish which temperature mode is superior in the context of aortic arch surgery.

## 4. Pharmacological Strategies of Neuroprotection

Pharmacological neuroprotection is based on the notion to reduce cerebral oxygen demand, limit the extent of cerebral damage during circulatory arrest, and, ultimately, reduce the incidence and severity of neurological deficits and neurocognitive dysfunction after aortic surgery. Many different strategies coexist, while some centers incorporated the administration of specific substances in their routine in contrast to others [29]. In the following section, different agents, their effectiveness, and current evidence are discussed. Table 2 summarizes pharmacological agents and exemplary studies for cerebral protection during aortic arch surgery.

### 4.1. Corticosteroids

Due to their anti-inflammatory properties, steroids have been investigated as a promising neuroprotection agent, but have failed to show a clear benefit in HCA animal models [30]. Furthermore, a large Cochrane review has not found sufficient evidence regarding neuroprotection in the setting of ischemic stroke [31]. Additionally, steroids are associated with neurotoxic properties and induce brain atrophy and hyperglycemia [32].

### 4.2. Magnesium

A systematic review has shown that magnesium improves the functional neurological outcome in the setting of cardiac arrest and cardiac surgery [33], although an RCT conducted by Mathew et al. has not shown improved neurocognitive function after cardiac surgery [34]. Another RCT found that magnesium improves neurologic function in the early postoperative period, but, after 3 months, there was no difference compared to placebo [35]. Although there is conflicting evidence regarding the actual benefit of magnesium in cardiac surgery, the application has been proven as a safe method [36].

### 4.3. Mannitol

Mannitol is an established method for reducing cerebral swelling and neurological dysfunction. Krüger et al. have shown that mannitol reduces the 30-day mortality from 18.7% to 8.9% using observational data from the German registry for Acute Aortic Dissection Type A (GERAADA) [36]. However, they did not observe a reduction in the mortality-corrected PND rates in the mannitol group.

### 4.4. Lidocaine

A PRISMA review evaluating the use of Lidocaine as neuroprotection has analyzed 6 RCTs and 963 patients and has concluded that it may have benefits regarding improved cognitive function in the early postoperative period, although limited by an insufficient level of evidence [37]. Ultimately, more studies investigating stroke and ischemic events are needed to determine whether Lidocaine is a potent neuroprotective agent [38].

### 4.5. Barbiturates

Although barbiturates such as thiopental seem like effective neuroprotective agents because they theoretically reduce cerebral activity, the lack of evidence in the setting of aortic surgery and, predominantly, the major and well-established side effects of barbiturates limit their use [38].

### 4.6. Propofol

In vitro studies have shown that propofol has neuroprotective properties by limiting apoptosis in hippocampal neurons [39], and experimental data from Kumagai et al. have shown that the intra-aortic injection of propofol prevents spinal cord injury during aortic surgery [40]. Controversially, Roach et al. have concluded that propofol does not facilitate a long-term reduction in neurologic dysfunction in the setting of valve surgery [41].

### 4.7. Inhaled Anesthetics

Although inhalative anesthetics such as isoflurane have been proven to have neuroprotective properties in animals and in vitro [42], clinical data in humans are controversial. Jovin et al. concluded that volatile anesthetics have failed to show benefit as part of neuroprotective preconditioning [38], but Chen et al. proposed in their meta-analysis that volatile anesthetics might be preferred over total intravenous anesthesia (TIVA) with substances such as propofol for cerebral protection [43].

### 4.8. Ketamine

Hudetz et al. have found that ketamine reduces neurocognitive dysfunction 1 week after cardiac surgery [44]. Subsequently, a large RCT has failed to show a reduction in postoperative delirium after major surgery. Furthermore, ketamine is known to cause hallucinations and nightmares, limiting its effectiveness [45].

### 4.9. Nitric Oxide

Nitric oxide did cause decreased neuroinflammation and apoptosis in animal studies [46], but evidence in humans is very limited and large randomized control trials are still missing. In summary, the use of pharmacological agents as a neuroprotection strategy remains highly controversial, with some evidence in the setting of general cardiac surgery but limited data on specific neuroprotection during circulatory arrest and no established consensus. Ultimately, large randomized trials are needed in order to further advance this aspect of neuroprotection and improve outcomes after aortic surgery.

## 5. Neuromonitoring

Various neuromonitoring strategies exist, with contradicting data and opinions. In the past, jugular bulb venous oxygen saturation monitoring was utilized frequently, but, due to its operational challenges and its invasive nature, it became widely replaced by other methods such as NIRS and EEG [47].

### 5.1. NIRS

Near infrared spectroscopy (NIRS) utilizes the regional hemoglobin oxygen saturation (rSO_2_) as a surrogate for cerebral perfusion [48]. The most recent guidelines recommend the use of bilateral NIRS and recommend algorithmic approach to evaluate and treat desaturation events [49]. The current literature does not provide a clear agreement on normal values or a certain threshold at which treatment or changes in management should be initiated. Moreover, conflicting evidence exists regarding the effectiveness of NIRS utilization in reducing neurological events in the setting of aortic surgery [48,50]. Furthermore, NIRS has also been used to determine optimal flow rates during selective antegrade cerebral perfusion. Friess et al. demonstrated that a cerebral flow rate of 8 mL/kg/min is more effective at reaching baseline regional cerebral oxygen saturation levels than a flow rate of 6 mL/kg/min, and that increasing the flow rate to 10 mL/kg/min does not yield further advantages [51].

### 5.2. EEG and SSEPs

Electroencephalography (EEG) is a widely used tool for intraoperative neurophysiological monitoring. Multichannel EEG has been partially replaced by the bispectral index (BIS) method, which assesses the frontal brain regions and extrapolates to evaluate the posterior regions. However, EEG serves as a surrogate for brain metabolic activity and becomes isoelectric during hypothermic circulatory arrest, rendering it ineffective for assessing the efficacy of perfusion techniques during circulatory arrest [52]. EEG is often used in conjunction with somatosensory evoked potentials (SSEPs) for dual neurophysiological monitoring, demonstrating a high negative predictive value for postoperative neurological adverse events and mortality [53]. Despite these positive results, conflicting evidence exists. Ghincea et al. did not find a statistically significant difference in outcomes with or without intraoperative neurophysiological monitoring [54].

Ultimately, no single method stands out as the best for assessing cerebral perfusion during aortic arch surgery, as studies present conflicting results. Further research is needed to resolve this issue.

### 5.3. Alpha-Stat vs. pH-Stat

Another crucial question arising in the management of patients undergoing aortic arch repair with hypothermia is whether blood gas and pH are monitored using the alpha-stat or pH-stat method. Briefly, alpha-stat maintains the patient’s pH at 7.40 at 37 °C (normothermic conditions) without adjusting for temperature changes, ensuring cellular homeostasis and enzymatic function. In contrast, pH-stat adjusts the oxygenator gas by adding CO_2_ to keep the blood pH at 7.40 according to the patient’s current body temperature, thus improving cerebral blood flow and cooling efficiency [55].

An ongoing controversy exists as to which method should be used in patients undergoing aortic arch repair. Advocates of the alpha-stat methods argue that cerebral autoregulation is preserved and that brain protection against ischemic events is superior [55]. Controversially, the pH-stat method enhances oxygen availability and allows for extended durations of hypothermic circulatory arrest [56]. Moreover, evidence, including one randomized controlled trial, indicates that this approach improves outcomes in infants [57]. A comprehensive review of 16 studies by Abdul Aziz et al. found that pediatric patients tend to benefit more from the pH-stat method, while alpha-stat is generally more advantageous for adult patients undergoing cardiac surgery [55]. A recent survey revealed that 87.9% of centers employ the alpha-stat method and 10.3% utilize the pH-stat method for managing mild to moderate hypothermia. In contrast, for deep to profound hypothermia, 56.9% of centers prefer the alpha-stat approach, 22.4% opt for pH-stat, and 20.7% adopt a dual strategy [58]. Ultimately, there is no definitive answer as to which pH management method is superior. More studies are necessary, particularly given the increasing use of mild to moderate hypothermia in aortic surgery.

## 6. Future Perspectives

### 6.1. Cerebral Monitoring

Transcranial Doppler sonography (TCD) has been developed as an alternative to other neuromonitoring methods such as electroencephalography (EEG) and near-infrared spectroscopy (NIRS). TCD has been shown to be a feasible monitoring device in the context of aortic arch surgery [59]. Most recently, Shah et al. have presented their experience using a new robotic transcranial Doppler device, which has allowed them to adjust the cerebral flows to reduce postoperative stroke and delirium [60]. These novel technologies have the potential to further improve neurological outcomes after aortic arch surgery in the future.

### 6.2. Biomarkers

Multiple biomarkers have been proposed to assess the risk of cerebral complications in the setting of aortic arch surgery and acute type A dissection. Most recently, NSE and S100B have shown potential to predict neurological complications after ATAAD surgery. While these parameters may not necessarily prevent neurological adverse events, further research on biomarkers can help improve the understanding of neurological injury during aortic arch surgery and aid in the development of new cerebral protection strategies [61].

### 6.3. Enhanced Recovery after Surgery (ERAS)

Implementing ERAS protocols has led to significant improvements in postoperative patient outcomes and enhanced functional status following general cardiac surgery [62]. However, further research is needed to develop tailored protocols for patients undergoing aortic arch surgery, particularly those at risk of neurological complications.

### 6.4. Visualization and Augmented Reality

Multiple novel technologies have been introduced to use Virtual Reality (VR) in aortic and vascular surgery. VR has proven applicability in the preoperative planning of open aortic arch procedures [63] and in intraoperative navigation and simulation during endovascular aortic procedures. Multiple head-mounted displays and smart glasses have been proposed to aid visualization during open and endovascular surgery, as well as in simulation training [64]. Case-specific simulation using VR, or some form of augmented reality, has the potential to improve the preparation of the physician for the planned procedure, thus avoiding complications and reducing surgical times, which might further facilitate neurological outcomes.

### 6.5. AI and Machine Learning

Machine learning (ML) is a promising innovation that has vast applications in medicine and surgery. Most recently, Nedadur et al. have used ML successfully to simulate how different cannulation strategies and temperatures would affect individual patient outcomes, such as death and the risk of stroke, in aortic arch surgery [65]. Machine learning has the potential to serve as a valuable tool for surgeons, aiding the decision-making process before and during complex aortic arch procedures.

### 6.6. Endovascular and Hybrid Approaches

The advent of endovascular procedures has resulted in the growing application of endovascular techniques for treating pathologies of the thoracic aorta as well as the aortic arch. Endovascular therapies have been used particularly in selected patients that are unsuitable or at high risk for open aortic arch surgery [66]. A growing number of studies explore the anatomical suitability of stent grafts in aortic arch pathologies, which range between 30.9% for elective repair of thoracic aneurysms [67] and 4–21% in acute type A aortic dissection [68]. The anatomical suitability for the Endo–Bentall procedure, which utilizes valve-carrying conduits, ranges from 31% to 80% [68].

Additionally, the use of endovascular techniques in hybrid procedures enables different treatment concepts to be applied. This ultimately leads to reduced circulatory arrest times, which positively impacts neurological outcomes. Novel therapeutic strategies such as Zone 2 aortic arch replacement with subsequent single-branch TEVAR positioned in the newly created landing zone reduce technical complexity and procedural duration.

Nevertheless, open repair remains the gold standard in the treatment of aortic arch pathologies, both in an emergent and elective setting [49,69].

## 7. The Vienna Approach

**Figure 1:**
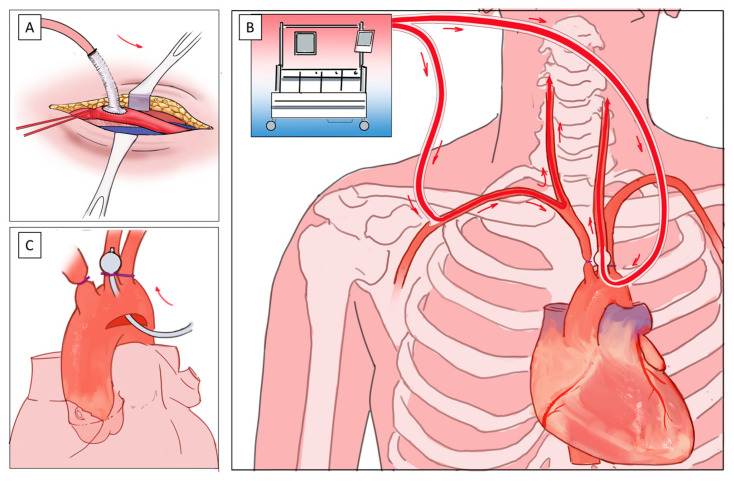
The Vienna approach for perfusion during aortic arch surgery: (**A**) Cannulation of the right axillary artery via an 8 mm side graft; (**B**) Bi-hemispheric antegrade cerebral perfusion during circulatory arrest via the right axillary artery (retrograde); and (**C**) A selectively introduced perfusion catheter in the left carotid artery.

Based on the best available evidence, our institution follows a standardized approach for cerebral protection in elective and acute aortic arch procedures. Every elective and almost all emergent patients undergoing arch procedures undergo preoperative computed tomography angiography, including the intracranial vessels, the supra-aortic branches, the complete aorta, and the iliac and femoral vessels (fast-acquisition CTA protocols to avoid motion artefacts are preferred). In addition to standard invasive hemodynamic monitoring, regional cerebral oximetry is used in order to identify cerebral desaturation events in a timely manner. When possible, the right axillary artery is the primary choice for arterial cannulation using an 8 mm graft with distal snaring of the vessel for perfusion control (Figure 1A). Following the initiation of cardiopulmonary bypass, the patient is cooled down to the desired temperature. In the case of total arch replacement (including further downstream adjuncts such as the frozen elephant procedure), we aim for 28 °C core temperature, whereas, in partial arch replacement (Zone 1–2 repairs) and Hemiarch procedures, the patient is cooled to 30–32 °C. When the target temperature is reached and all supra-aortic vessels are identified and ensnared, the patient is put in the Trendelenburg position, the pump flow is reduced to approximately 1.5 L/min (depending on patient body surface area), and the innominate artery is cross-clamped (Figure 1B). The aorta is incised and, immediately, a perfused dedicated balloon occlusion catheter is inserted carefully into the right carotid artery to establish bi-hemispheric antegrade cerebral perfusion (Figure 1C). After the completion of the procedure, cannulas are removed, the pump flow is increased, and meticulous deairing is performed while the patient is rewarmed.

**Figure 2:**
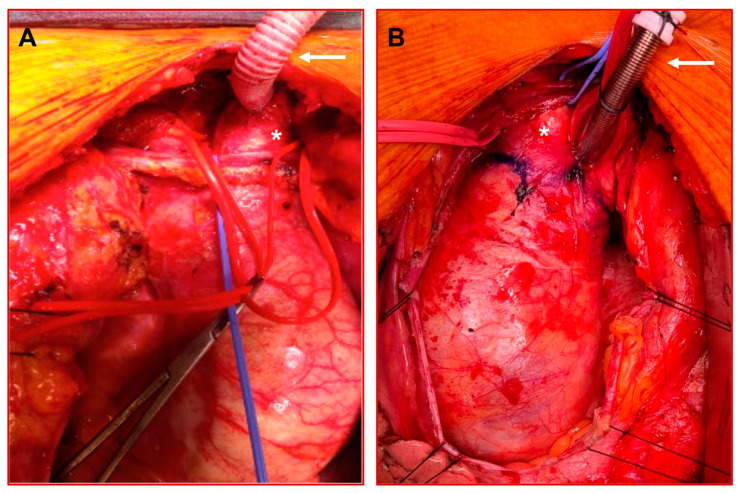
Alternative cannulation techniques in arch surgery: (**A**) Cannulation of the innominate artery (*) via an 8 mm side graft (arrow); (**B**) direct arch cannulation above the level of the innominate artery (*) via the Seldinger technique with an arterial cannula (arrow) for establishment of cardiopulmonary bypass.

In the case of alternating patient anatomy, adaptions to the cannulation and perfusion model can be applied utilizing alternative cannulations sites. For patients with a feasible anatomy of the innominate artery (length > 2 cm, no calcification or soft plaques), cannulation can be performed via an 8 mm graft, similar to the subclavian artery (Figure 2A). This obviates the patient from an additional incision and possible complications. In other cases, a direct arch cannulation might be performed for the establishment of cardiopulmonary bypass and the cooling of the patient, which can then be removed during circulatory arrest (Figure 2B).

Applying this approach allows for a near physiological cerebral perfusion while obviating the patient from deep hypothermia, even in the case of more complex procedures. Nevertheless, more evidence in this area is crucial, and prospective trials are needed to further improve outcomes and reduce cerebral complications in aortic arch surgery.

## 8. Limitations

This study has several limitations that must be acknowledged. The primary limitation is the lack of randomized controlled trials that compare different cerebral protection strategies. Most available data come from observational studies and registries, which are prone to bias. Additionally, there is variability in surgical techniques, perfusion methods, temperature management strategies, and patient populations across studies, making it difficult to draw definitive conclusions. Moreover, while novel techniques such as hybrid approaches and machine learning show promise, the evidence supporting these methods is still preliminary and based on small sample sizes. Pharmacological neuroprotection strategies have limited and conflicting evidence, often extrapolated from other contexts. Lastly, this study includes our institutional approach that may not be directly applicable to other settings, requiring adaptation to different patient populations and resources. Addressing these limitations through rigorous, standardized research in future studies is essential for advancing the field and improving patient outcomes.

## Figures and Tables

**Table 1:** Relevant and exemplary studies investigating cerebral protection during aortic arch surgery.

Author, Year	Study Type	Patient Collective	Cerebral Perfusion Technique	CA Time (Minutes)	Mean Temperature (°C)	Neurological Deficit (PND or TND Rates)	Conclusions
Piperata et al., 2022 [18]	Multi-center, retrospective	ATAAD n = 646 PSM analysis: n = 378	uACP: 39% bACP: 61% PSM analysis uACP n = 189 bACP n = 189	uACP: 34 (28–42) bACP: 37 (28–50)	uACP: 28 °C (28–28 °C) bACP: 27.5 °C (25–28 °C)	PND uACP: 4% bACP: 14% (*p* < 0.001) TND uACP: 11% bACP: 12% (*p* = 0.061)	- uACP and bACP are both valid brain protection strategies - PNDs are significantly less frequent in uACP - All combined-complications are significantly less frequent in uACP
Benedetto et al., 2021 [22]	National Adult Cardiac Surgical Audit, prospective	ATAAD n = 1929	uACP: 6.1% bACP: 39.4% RCP: 11.5% DHCA: 43%	37.2 +/− 28.5	n/a	uACP: 9.4% bACP: 14.6% RCP: 13.1% DHCA: 14.2% (p: n/a)	- uACP and bACP are superior to DHCA alone regarding death and CVA - uACP may be superior to bACP in short CA times
Angleitner et al., 2020 [3]	Single- center, retrospective	ATAAD n = 184	uACP: 51% bACP: 49%	34 (26–49)	n/a	PND uACP: 19.4% bACP: 18.7% (*p* = 0.753) TND uACP: 9.7% bACP: 7.7% (*p* = 0.226)	- bACP vs. uACP have similar neurological outcomes - bACP may be superior to uACP in perfusion durations > 50 minutes
Norton et al., 2020 [23]	Single-center, retrospective	ATAAD n = 307	uACP: 45.6% bACP: 54.4%	uACP: 29 (23–38) bACP: 45 (38–55)	bACP: 17 °C (16–18 °C) uACP: 20 °C (18–24 °C)	uACP: 6% bACP: 9% (*p* = 0.4)	- uACP and bACP are equally effective - uACP recommended for simplicity and less manipulation of arch branch vessels
O´Hara et al., 2020 [20]	STS Adult Cardiac Surgical Database, retrospective	ATAAD n = 6387	ACP: 46.2% RCP: 22.6% DHCA: 31.2%	ACP: 25 (26–48) RCP: 33 (25–45) DHCA: 26 (20–34)	ACP: 22 °C (18.4–25) RCP:17.6 °C (19–21.9) DHCA: 18.8 °C (17.7–21.4)	ACP: 12.5% RCP: 11.2% DHCA: 13% (*p* = 0.06)	- Cerebral perfusion techniques such as ACP and RCP are associated with reduced death and stroke risk
Okita et al., 2015 [21]	Japan Adult Cardiovascular Surgery Database, retrospective	Arch repair excl. ATAAD All n = 8169 PSM analysis n = 2282	DHCA/RCP: n = 1141 (14%) ACP: n = 7038 (86%) PSM analysis: DHCA/RCP: n = 1141 ACP: n = 1141	n/a	ACP: 24.2 °C +/− 3.2 °C DHCA/RCP: 21.2 °C +/− 3.7 °C	uACP: 6.7% bACP: 8.6% (*p* = 0.83)	- HCA/RCP and ACP have comparable outcomes (death, stroke, reoperation) - Longer ICU stays in HCA/RCP group - ACP might be preferred for complicated aortic arch procedures
Zierer et al., 2012 [24]	Multi-center, prospective	Aortic Arch replacement incl. ATAAD (35%) n = 1002	uACP: 33% bACP: 67%	36 +/− 19	28–30 °C	PND uACP: 2% bACP: 4% (*p* = 0.6)	- uACP offers at least equal brain protection as bACP
Kruger et al., 2011 [19]	German Registry for Acute Aortic Dissection Type A (GERAADA)	ATAAD n = 1558	DHCA: 22.8% RCP: 2.2% uACP: 40.3% bACP: 29.1%	DHCA: 22.7 +/− 14.3 RCP: n/a uACP: 32.2 +/− 17.9 bACP: 37.4 +/− 23.6	n/a	PND HCA: 14.9% uACP: 12.6% bACP: 14.1% (p: n/a)	- Similar results with times < 30 min - During Longer periods ACP is advisable - uACP and bACP are equivalent

Abbreviations: PSM = Propensity Score Matching, uACP = unilateral antegrade cerebral perfusion, bACP = bilateral antegrade cerebral perfusion, RCP = retrograde cerebral perfusion, ATAAD = acute type A aortic dissection, (D)HCA = (deep) hypothermic circulatory arrest, CVA = cerebrovascular accident, PND = permanent neurological deficit, TND = temporary neurological deficit.

**Table 2:** Relevant pharmacological agents and exemplary studies for cerebral protection during aortic arch surgery.

Agent	Author, Year	Conclusions
Corticosteroids	Manetta et al., 2018 [30], Sandercock & Soane 2011 [31], Sapolsky 1996 [32]	- Investigated for neuroprotection but failed in HCA animal models. - Cochrane review could not find sufficient evidence in ischemic stroke. - Associated with neurotoxicity.
Magnesium	Pearce et al., 2017 [33], Mathew et al., 2013 [34], Bhudia et al., 2006 [35]	- Improves neurological outcomes in cardiac arrest and -surgery. - Conflicting evidence regarding actual benefit but proven safe.
Mannitol	Kruger et al., 2013 [36]	- Reduces cerebral swelling. - Reduced 30-day mortality from 18.7% to 8.9% in GERAADA registry
Lidocaine	Hung et al., 2022 [37], Jovin et al., 2019 [38]	- May improve cognitive function early post-op. - More studies needed.
Barbiturates	Jovin et al., 2019 [38]	- Theoretical benefits but major side effects limit use.
Propofol	Li et al., 2018 [39], Kumagai et al., 2006 [40], Roach et al., 1999 [41]	- Shows neuroprotective properties in experimental studies. - Mixed clinical results.
Inhaled anesthetics	Bickler et al., 2005 [42], Chen et al., 2017 [43]	- Proven neuroprotective in animals. - Controversial human data.
Ketamine	Hudetz et al., 2009 [44], Avidan et al. 2017 [45]	- Reduces neurocognitive dysfunction early post-op. - Side effects limit use.
Nitric Oxide	Linardi et al., 2021 [46]	- Decreases neuroinflammation in animal studies. - Limited human evidence.

Abbreviations: HCA = (deep) hypothermic circulatory arrest.
